# Diagnostic Accuracy of Nipple Aspirate Fluid Cytology in Asymptomatic Patients: A Meta-analysis and Systematic Review of the Literature

**DOI:** 10.1245/s10434-020-09313-9

**Published:** 2020-11-09

**Authors:** Natasha Jiwa, Rishikesh Gandhewar, Hemali Chauhan, Hutan Ashrafian, Swathica Kumar, Corrina Wright, Zoltan Takats, Daniel Richard Leff

**Affiliations:** 1 Department of Surgery and Cancer, Imperial College London, London, UK; 2 Royal College of Surgeons Ireland, Dublin, Ireland; 3 Imperial College Healthcare Trust, London, UK

## Abstract

**Purpose:**

To calculate the diagnostic accuracy of nipple aspirate fluid (NAF) cytology.

**Background:**

Evaluation of NAF cytology in asymptomatic patients conceptually offers a non-invasive method for either screening for breast cancer or else predicting or stratifying future cancer risk.

**Methods:**

Studies were identified by performing electronic searches up to August 2019. A meta-analysis was conducted to attain an overall pooled sensitivity and specificity of NAF for breast cancer detection.

**Results:**

A search through 938 studies yielded a total of 19 studies. Overall, 9308 patients were examined, with cytology results from 10,147 breasts [age (years), mean ± SD = 49.73 ± 4.09 years]. Diagnostic accuracy meta-analysis of NAF revealed a pooled specificity of 0.97 (95% CI 0.97–0.98), and sensitivity of 0.64 (95% CI 0.62–0.66).

**Conclusions:**

The diagnostic accuracy of nipple smear cytology is limited by poor sensitivity. If nipple fluid assessment is to be used for diagnosis, then emerging technologies for fluid biomarker analysis must supersede the current diagnostic accuracy of NAF cytology.

**Electronic supplementary material:**

The online version of this article (10.1245/s10434-020-09313-9) contains supplementary material, which is available to authorized users.

Methods for early breast cancer detection enable lesions to be treated at the earliest possible time point, increasing survival and improving patient outcomes (e.g. lumpectomy versus mastectomy). Early identification impacts surgical treatment plans. This is exemplified by breast cancer clinical outcome measures data, which indicate that a greater proportion of screening cancers undergo breast conserving surgery when compared with patients who present symptomatically.^1^

The breast screening programme, however, has certain limitations. Screening is conducted once every 3 years in the UK and only detects two-thirds of all breast cancers that arise during the 3-year interval.^2^ Therefore, one-third of patients will present with ‘interval breast cancers’ between two screening mammograms and half of these occur in the 3rd year after screening.^2^ One in 2500 screened women will have a missed cancer.^3^ The reasons for missed cancers include breast density, absence of the radiographic classification of cancer or subtle radiographic signs (often seen in invasive lobular carcinomas), poor technique and misinterpretation.^4^^,^^5^ Digital mammography has a sensitivity of 89% but a specificity of only 72%.^6^ High-risk women undergo more frequent mammograms, which carries radiation risk, or regular MRIs, which have a number of contra-indications as well as a cost implication.^7^ In addition, data on screening uptake indicates that uptake to the screening programme has been decreasing over the last 10 years, approaching the acceptable 70% mark.^8^

As we move into an era of personalised screening, we need techniques that better estimate an individual’s breast cancer risk. Whilst models for estimating breast cancer risk do exist, such as the Gail,^9^ Claus 10 and Tyrer–Cuzick models,^11^ they are not without limitations.^12^^–^14 This is primarily due to limited discrimination accuracy.^15^ The Gail model, for example, tends to underestimate risk as it only takes into account first-degree relatives and does not consider age of onset of cancer.^11^^,^^16^ The Claus model, although accounting for many of the genetic variations of risk, does not consider any non-hereditary risk factors and reflects women in the USA in the 1980s, mirroring a population with an outdated incidence of breast cancer^16^ in both North America and Europe.^17^^,^^18^ Although the Tyrer–Cuzick model adjusts for many of these genetic factors, it focuses predominantly on penetrance of BRCA1/2 mutations rather than the plethora of non-BRCA mutations^14^^,^^19^ and tends to over-estimate risk in women with atypical hyperplasia.^13^ As has been suggested with breast density,^15^ this begs the question as to whether adding further adjuncts such as nipple fluid cytological findings, if predictive findings are significant, could strengthen current risk assessment models and offer a personalised approach to risk screening in the future.

Cytopathology was invented and utilised in the 1920s by George Papanicolaou for early diagnosis of reproductive tract cancer, where he was able to readily distinguish between normal and malignant cells in the cervix.^20^ Cytological evaluation of breast tissue has been ongoing since the 1950s,^21^ illustrating its cellular composition using a five-tiered classification.^22^ Its potential as a screening tool for nipple fluid and has been evaluated by several groups.^23^^–^28 However, the evidence base for the relative risk of breast cancer following abnormal cytology comes largely from breast tissue fine needle aspirate cytology or breast biopsies.^29^^–^31 Whether the two can be compared is controversial. Atypia in nipple fluid is different to ADH in tissue biopsies, for example. Atypia is more suggestive and can be due to degenerative cellular changes, whereas ADH in tissue biopsies is based on established links to cancer from long-term follow-up studies.^32^

With the growth of metabolomics over the past decade, the study of nipple aspirate fluid has expanded to investigate the presence and quantities of a variety of substances, including proteins, lipids, DNA and the microbiome.^33^^–^36 This has allowed detailed evaluation of nipple fluid biomarkers, from which personalised risk screening is possible. However, nipple fluid cytology is the starting point for all future tests. As the current gold standard clinical test, information about both its diagnostic capabilities and predictive validity are vital. To the authors’ knowledge, a meta-analysis of studies containing diagnostic data on the accuracy of nipple aspirate fluid obtained by various methods has not been conducted to date. With the explosion of tools available for examining nipple fluid, and the relatively small number of studies that report the predictive capability of abnormal cells in nipple aspirate fluid, these data are more important than ever in the assessment of nipple fluid’s diagnostic and screening potential.

The aim of this study was to systematically review the published literature to evaluate the diagnostic accuracy of nipple aspirate fluid cytology, against which the performance of new and emerging diagnostic and screening technologies may be compared.

## Methods

### Literature Search

The literature review was conducted as per guidelines for the ‘Preferred Reporting Items for Systematic Reviews and Meta-analyses’ (PRISMA). The literature search was conducted using MEDLINE, EMBASE and SCOPUS databases. Relevant studies were identified using MeSH terms of key phrases from the research question (up to date as of October 2019). They were specific to ‘nipple aspirate fluid’ in various formats AND ‘cytology’ in its various forms. The following Medical Subject Headings (MeSH) and key words were used in combination with AND/OR operators: ‘nipple aspirate fluid’ OR (‘breast’ OR ‘nipple’ adjacent to ‘secretion*’ OR ‘aspirat*’) with (‘cytodiagnosis’ OR ‘cytolog* OR ‘papanicolaou’ OR ‘cytodiagnos*’).

### Inclusion and Exclusion Criteria

Title and abstract review was conducted according to the pre-defined inclusion and exclusion criteria as detailed below, for each part of the review.

### Inclusion Criteria

Studies reporting data on the diagnostic potential of the cytology of nipple fluid were included in the review. Both radiological and tissue histopathological confirmation of diagnosis were accepted as diagnostic modalities for validation of breast cancer. All methods of retrieving nipple fluid, including ductoscopy and ductal lavage were included, as well as those of various study designs, i.e. asymptomatic/high-risk patients and those with a known diagnosis of cancer. Only studies with full text available in the English language on human subjects, until August 2019 were included.

### Exclusion Criteria

Articles were excluded by title and abstract screening if they were review articles or carried no original/primary data, had an irrelevant research question or involved processing fine needle aspirate cytology (FNAC) of breast tissue rather than the nipple aspirate fluid itself. Papers that were conducted outside a clinical environment were excluded, as well as studies without histopathological or follow-up radiological data to confirm cytological diagnoses. Diagnostic accuracy data such as true positive, true negative, etc. were not compulsory and were calculated from the raw data provided where possible.

### Study Quality

Study quality was evaluated using the ‘Quality Assessment of Diagnostic Accuracy Studies 2’ (QUADAS-2) scoring system checklist,^37^ and was conducted by two independent investigators (NJ and RG). All the QUADAS-2 questions were included in the scoring, providing a maximum score 14.^37^ Each question was given a score of 0, 1 or 2 depending on whether the question was not answered, unclearly answered or fully answered. To consider the study accurately conducted and analysed, the studies had to report the type of patient included in the study (symptomatic, asymptomatic, high-risk or post-operative). The cytopathologist interpreting the results had to be defined (i.e. 1 or 2 pathologists; independently reporting) and it had to be stated whether they were blinded to the clinical results.

### Data Collection

Independent assessment by two investigators (NJ and RG) was conducted using Covidence (Veritas Health Innovation, Melbourne, Australia) systematic review software.^38^ Any conflicts were discussed and resolved with explanations of ‘yes’, ‘no’ or ‘uncertain’. All ‘uncertain’ cases underwent full-text screening: justification for inclusion or exclusion was documented within the system (see supplementary table) and was discussed with senior authors (HA and DRL). Demographic and accuracy data from the included studies was recorded using a pre-defined Excel spreadsheet. In particular, data was collected on: (1) first author and year of publication; (2) number of patients; (3) true positives; (4) true negatives; (5) false positives; (6) false negatives; (7) average age of patient; (8) total QUADAS-2 scoring; (9) method of collection; (10) sensitivity; (11) specificity and (12) positive predictive values. Following data extraction, studies were subdivided by their method of collection, e.g. ductal lavage, manual compression etc. for subsequent sub-analysis of sensitivity and specificity by method. Sensitivity and specificity data were calculated to 2 decimal points. Median ± IQR age (years) was recorded where available, otherwise mean ± SD age was used.

### Meta-analysis

A bivariate and hierarchical model was used to calculate the overall diagnostic accuracy of the studies included in the meta-analysis. This allowed identification of any statistical differences between the models and provided an internal cross-reference for results produced. The sensitivity and specificity of the results was assessed using a hierarchical summary receiver-operating characteristic (HSROC) model. Pooled diagnostic sensitivity and specificity was calculated using 11 and 19 of the studies alike (all studies with sensitivities of 0 were excluded). A diagnostic HSROC curve was produced for 6 studies from within the 19 included and this allowed demonstration of the diagnostic performance of cytology of nipple aspirate fluid. For utilisation of this model, studies containing the values ‘0’ and ‘1’ were excluded. The trapezoid rule was utilised to calculate the pooled area under the curve. In the model, a value 1.0 indicates a perfect test with 100% accuracy and 0.5 indicates that the test is equally likely as it is unlikely to be true.

## Results

### Malignant Diagnostic Cytology

**Fig. 1:**
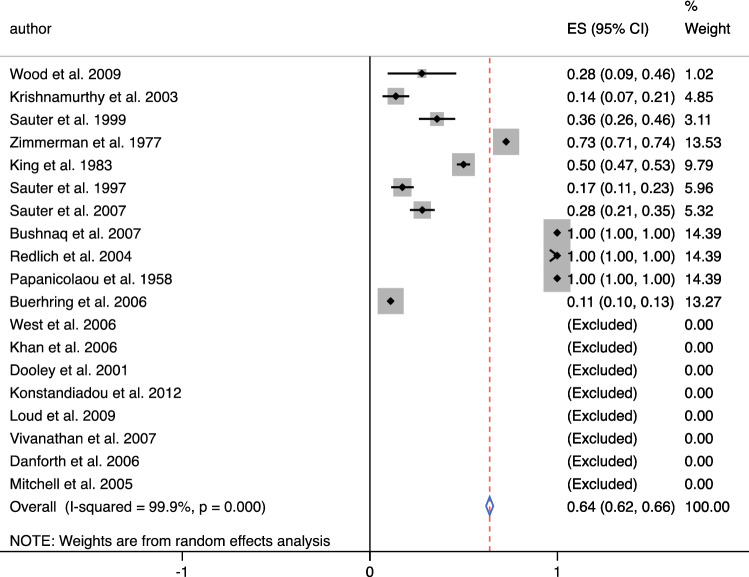
ES, effect size; 95% CI, 95% confidence interval; % weight, percentage weight carried by the study calculated from the random effects analysis. Overall sensitivity from 11/19 studies 0.64 [0.62–0.64]

**Fig. 2:**
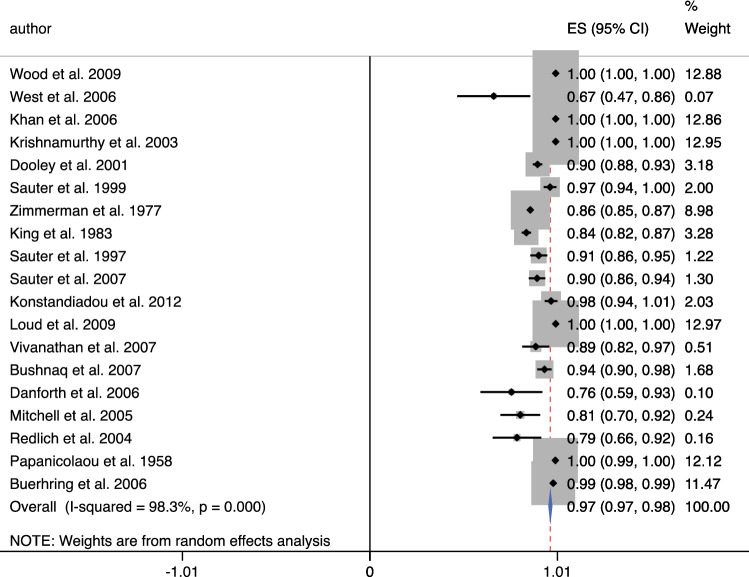
ES, effect size; 95% CI, 95% confidence interval; % weight, percentage weight carried by the study calculated from the random effects analysis. Overall specificity from 19/19 studies 0.97 [0.97–0.98]

**Fig. 3:**
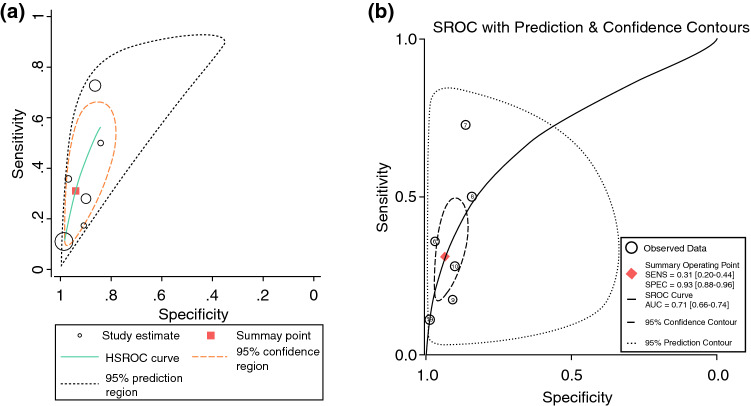
**a** Meta-analysis HSROC curve for cytology and **b** prediction and confidence contours for the six studies included in the meta-analysis. The *x*-axis demonstrates 1-specificity (the true negatives) and the *y*-axis demonstrates the sensitivity (the true positives). The curve delineates the true positive rate of nipple cytology at each true negative value. A perfect test is one in which the results are closest to 1, i.e. 100% accuracy

A total of 19 studies 24^,^^39^^–^56 were included in the diagnostic arm of the systematic review. These all contained clinical data on the diagnoses acquired from NAF cytology, which were correlated with either imaging or histology following a biopsy. From this, sensitivity and specificity data were either extracted or calculated. Results for 19 studies that met the criteria are included in Table 1. The publication dates included in these studies ranged from 1958 to 2009, statistics summarised in Table 2. Mean or median age was available for 16 of the 19 studies with overall ages ranging from 20 to 87. The mean or median ages ranged from 40.3 to 57.0. The calculated true positives, true negatives, false positives and false negatives, as well as the sensitivity, specificity and positive predictive value for each study are also included in Table 1. The overall sensitivity of cytology was calculated to be 0.64 (95% CI 0.62–0.66) (Fig. 1) and the specificity was 0.97 (95% CI 0.97–9.98) (Fig. 2). The diagnostic accuracy curve for these studies is illustrated in Fig. 3a, b.

**Table 1:** Demographics and outcome data—studies containing diagnostic data for meta-analysis

Author, year	n	tp	tn	fp	fn	age	qtot	meth	sens	spec	Ppv
Wood et al. 2009	23	5	18	0	13	51.0	10	1	0.3	1.0	1.0
West et al. 2006	22	0	8	4	0	51.5	12	1	0.0	0.7	0.0
Khan et al. 2005	20	0	7	0	5	54.2	12	1	0.0	1.0	0.0
Krishnamurthy et al. 2003	91	5	8	0	31	54.0	10	2	0.1	1.0	1.0
Dooley et al. 2001	507	0	370	40	0	51.9	8	5	0.0	0.9	0.0
Sauter et al. 1999	95	14	32	1	25	52.0	10	3	0.4	1.0	0.9
Zimmerman et al. 1977	4685	8	190	30	3		8	4	0.7	0.9	0.2
King et al. 1983	796	20	16	3	20		6	3	0.5	0.8	0.9
Sauter et al. 1997	152	4	20	2	19	51.7	14	3	0.2	0.9	0.7
Sauter et al. 2007	177	28	54	6	72		8	3	0.3	0.9	0.8
Konstandiadou et al. 2012	80	0	78	2	0	45.8	10	1	0.0	1.0	0.0
Loud et al. 2009	171	0	92	0	92	40.3	12	1	0.0	1.0	0.0
Visvanathan et al. 2007	69	0	25	3	0	46.6	10	1	0.0	0.9	0.0
Bushnaq et al. 2007	150	10	264	17	0	48.0	8	1	1.0	0.9	0.4
Danforth et al. 2006	25	0	16	5	0	57.0	6	1	0.0	0.8	0.0
Mitchell et al. 2005	52	0	17	4	4	43.0	12	5	0.0	0.8	0.0
Redlich et al. 2004	37	1	19	5	0	51.7	10	1	1.0	0.8	0.2
Papanicolaou et al. 1958	412	1	607	1	0	47.3	8	2	1.0	1.0	0.5
Buerhring et al. 2006	744	1	529	7	8		12	5	0.1	1.0	0.1

*N*, number of patients; *tp*, true positives; *tn*, true negatives; *fp*, false positives; *fn*, false negatives; *qtot*, QUADAS total score; *meth*, method of collection of nipple fluid (*1*: ductal lavage, *2*: manual compression, *3*: manual pump, *4*: not specified, *5*: two methods combined); *sens*, sensitivity; *spec*, specificity; *ppv*, positive predictive value

**Table 2:** Summary statistics of the 19 studies included and overall diagnostic accuracy data of nipple aspirate fluid cytology

Summary statistics	
Total number of breasts: 10,147	Methods of collection: 11 DL; 6MP; 4MC; 3DT; 1NS
Total number of patients: 9308	Non-yielder: 30.5% ± 26.4 (mean)
Mean age: 49.73 ± 4.09 years	Insufficient sample: 38.87% (mean)

Parameter	Estimate	95% CI
Sensitivity^a^	0.64	0.621–0.659
Specificity^b^	0.971	0.966–0.977
Positive likelihood ratio	4.70	2.80–7.80
Negative likelihood ratio	0.74	0.63–0.87
Diagnostic odds ratio	6.00	4–11

Overall summary statistics from the studies included in the meta-analysis

*DL*, ductal lavage; *MP*, manual pump; *MC*, manual compression; *DT*, dual technique; *NS*, not specified

^a^11 of the 19 studies were utilised to calculate the overall sensitivity (studies that reported a sensitivity of 0 were excluded)

^b^All 19 studies were included to calculate the overall specificity

## Discussion

This meta-analysis assimilates the diagnostic accuracy of nipple aspirate fluid cytology across published clinical studies as well as the future risk of developing breast cancer depending on the type of previous nipple aspirate fluid cytology finding. The results reveal an overall low sensitivity of 0.64 (95% CI 0.62–0.66) and high specificity of 0.97 (95% CI 0.97–9.98), when NAF cytology is used as a diagnostic tool.

Despite the continued use of cytology for evaluating symptomatic nipple discharge fluid, NAF cytology has not successfully penetrated clinical practice as a method of assessment for asymptomatic women at high risk of breast cancer, or indeed as an adjunct to screening. The reasons for this may include both the limited diagnostic accuracy in this setting, as highlighted by our findings, as well as the challenges involved in collection of fluid, in particular the potential for non-producers, the fact that not all terminal duct-lobular units drain to the nipple,^57^ the proportion of smears deemed inadequate,^58^ and whether methods for NAF harvest are deemed acceptable by patients and the public.^59^

The primary finding of this meta-analysis is that the sensitivity of NAF cytology for cancer detection in asymptomatic women is poor [0.64 (95% CI 0.62–0.66)] and yet the specificity is extremely high [0.97 (95% CI 0.97–0.98)]. Overall, high specificity and low sensitivity data is echoed across individual studies and also in those presenting with symptomatic nipple discharge,^60^^,^^61^ with a diagnostic accuracy for malignancy of only 31%.^60^ The reason for such low sensitivity of nipple aspirate fluid is that discharge is often “physiological” in nature, has a generally high acellular or paucicellular composition,^58^^,^^62^^,^^63^ and therefore is thought to consist almost exclusively of background debris and/or proteinaceous material.^58^^,^^64^

Methods for NAF evaluation, whether they be cytological assessment or otherwise, suffer as a result of the relatively high proportion of “non-yielders”: women in whom techniques to obtain NAF fail. Statistics from all the studies included in this review demonstrate a non-yielding percentage of 30.5% ± 26.4% across the various methods of fluid collection of nipple aspirate fluid. The lowest non-yielders were those that underwent ductal lavage studies.^40^^,^^65^^,^^66^ However, ductal lavage is subject to selection bias, as those in whom cannulation for ductoscopy was not possible are unlikely to have been selected for ductal lavage. Similarly, a low non-yielder rate was observed in those that underwent expression using a manual pump.^46^^,^^66^^–^68 In these studies, non-yielders ranged from 0 to 5.9%, and three out of four of the studies utilised a modified breast pump.^67^^–^69 However, these samples were acquired from mastectomy specimens, immediately following its excision from the chest wall, rather than from the awake patient. This allowed for a reasonable amount of negative pressure to be applied to the nipple-areolar complex without concern of causing pain or discomfort. These figures, whilst likely to be an underestimation of non-yielding status using the same method in a patient who is conscious, provide reassuring evidence that physiological nipple aspirate fluid is present and easily accessible in patients under general anaesthetic.

One of the greatest limitations of nipple fluid cytology according to the results from the studies included in the current analysis, is the percentage of samples produced that are ‘inadequate’. Here we demonstrate a mean of 38.9% of analysed samples that were deemed inadequate. This can often be the case with ductal lavage samples, in which fluid content is diluted by saline. This has a major cost implication. If almost 40% of all samples processed are inadequate, and the cost of processing and reporting each slide is £75, then this represents substantial waste and ultimately reduces the value of the test. Repeating NAF assessments is also problematic. For example, ductal lavage may be painful 70 or difficult to tolerate under topical/local anaesthetic, and manual pumps and/or automated pumps may cause discomfort or skin surface irritation.^26^ In addition, the process of collecting and reporting smears is confounded by inappropriately prepared slides in the absence of a cytopathologist in the outpatient department, which can lead to sub-optimal slides due to air drying artefacts, contamination or inadequate fluid distribution. Reporting may be subject to inter-reporter variability or relative inexperience, as well as the presence of atypical cellular changes unrelated to a malignancy, leading to either a higher degree of false positives or false negative findings.

Next is the challenge of proportion of ducts whose biocomposition can accurately be evaluated within NAF. Most breast cancers arise from the epithelial lining of the terminal ducts—invasive ductal carcinomas. NAF therefore provides a mirror of what is occurring in the tumour microenvironment or, in high-risk individuals, in the lead-up to cancer.^71^ However, it has also been shown that not all ducts drain to the nipple surface,^57^ suggesting that even if NAF cytological analysis has superior diagnostic accuracy to that demonstrated here it could still miss a proportion of breast cancers.

As an important adjunct, the predictive validity of nipple aspirate fluid has been investigated by Wrensch and her group 72^–^76 at various time points between 1992 and 2010, using several patient groups recruited at different time points. Results have yielded an overall threefold increase in the risk of breast cancer with a NAF finding of atypia. Although findings are limited by a potential overlap in patient cohorts within the studies and therefore a duplication of datasets, a threefold increase in risk of developing breast cancer should not be overlooked. Long-term follow-up studies, such as that by Page et al.,^32^ highlight that detection of ALH/ADH has an associated 4–5 times risk of breast cancer. However, there is a relative scarcity of similar evidence with nipple smear cytology and even ductal lavage.^77^ Evidence tends to be almost completely extrapolated from FNAC studies with tissue^29^^–^32 and, in fact, it is this body of evidence which has supported commercial products such as HALO (NeoMatrix, Irvine, CA), the nipple aspiration device. In nipple fluid, atypia is more suggestive than diagnostic and can even be due to degenerative cellular changes. Its significance is therefore inconclusive and would have to be carefully considered prior to incorporation into existing and established models of risk prediction, such as the Gail model or Tyrer–Cuzick. Integrating a clinical assessment tool into an existing model could both strengthen and personalise risk in asymptomatic, high-risk women, as illustrated by Vilmun et al.^15^ when reviewing the impact of adding breast density to breast cancer risk models.^78^ In order to offer a personalised approach to risk screening, implementation of a stratification protocol offering varying screening regimes according to their elicited risk, following the interrogation of nipple smear cytology, would be required to be undertaken in various patient cohorts. This would be undertaken with a view to a reduction in the diagnosis of interval cancers, an increase in the diagnosis of early breast cancer and a reduction in mortality. For example, the “WISDOM trial”^79^ was a multi-centre randomised controlled trial that allowed for both risk-based and observational screening. It takes into account personal and genetic risk, including mutations such as BRCA1/2, ATM, CDH1, CHEK2, PALB2, PTEN, STK11 and TP52, as well as a polygenic risk score from 96 lower-risk common genetic variants (SNPs) with known association with breast cancer, and an updated polygenic risk model, including ethnicity- and race-specific SNPs that are shown to confer risk, to calculate a personalised risk score.

Moreover, with promising early data emerging from the interrogation of nipple fluid using innovative technologies such as mass spectrometry, as well as the known limitations of cytology, it is reasonable to suggest that various metabolites found within nipple fluid have great diagnostic potential. With the growth of metabolomics over the past decade, the study of nipple aspirate fluid has expanded to investigate the presence and quantities of a variety of substances, including proteins, lipids, DNA and the microbiome. Proteomic analysis of nipple aspirate fluid works on the principle that it contains a concentrated source of proteins from cancerous ducts, which may identify tumour-specific protein patterns.^80^ In 2004, Alexander et al.^81^ identified candidate markers using matrix-assisted laser desorption ionization time-of-flight (ESI Q-TOF) proteomic analysis and validated the markers identified using quantitative, high-throughput ELISA analysis. Among their subjects, GCDFP-15 levels were lower and AAG levels correlated with presence and stage of breast cancer disease. Similarly, in 2007, He et al.^82^ identified a set of 8 protein markers which collectively gave a 63% sensitivity, 89% specificity and 76% accuracy for distinguishing between cancer and normal pathologies.^82^ Further work on the proteome was conducted by Pavlou et al.^36^ in 2010. The authors utilised LCMS-MS to generate an extensive nipple aspirate fluid proteome identifying over 800 unique proteins, more than 50% of which were extracellular plasma membrane proteins. In 2017 Shaheed et al. 83 used manual expressing techniques to acquire NAF samples for proteomic analysis with 2D LC–MS separation. The results demonstrated that in the majority of individuals with bilateral samples, paired samples illustrated protein profiles that were similar. They reported an average of 1374 proteins per sample with significant progress, identifying 1374 new proteins from those previously seen by Pavlou et al. 36 in NAF. In terms of lipidomics, a study by Matos Do Canto et al. 33 in 2016 identified up to 83 ions with a fold change — metabolites included endogenous metabolites such as amino acid derivatives, products of lipid metabolism, glycerophospholipids and phosphatidylserine. It is the first known study that demonstrates the feasibility of conducting a comprehensive metabolomic profiling of breast tumours using ductal lavage. Due to the contemporaneous nature of these studies, the process of biomarker development is still in its early stages and is therefore a promising field of study.

## Conclusion

The current systematic review and meta-analysis provides new diagnostic accuracy data for nipple aspirate fluid cytology, including pooled data overall, whilst taking into account the collection method. The results demonstrate that the diagnostic accuracy of nipple fluid cytology is limited due to poor sensitivity secondary to a paucicellular material. Emerging techniques for surveillance and screening of patients who carry a risk of breast cancer will need to have a personalised approach and surpass the present diagnostic accuracy of cytology, whilst taking into account cost effectiveness, reproducibility of results, user dependency and turn-around time in the laboratory. The sensitivity and specificity should be high enough to warrant further assessment in the form of imaging or a confirmatory biopsy (histopathology).

## Electronic supplementary material

Below is the link to the electronic supplementary material.

Supplementary material 1 (DOCX 12 kb)

Supplementary material 2 (PDF 33 kb)
