# Supplementary material for: Diagnostic Accuracy of Nipple Aspirate Fluid Cytology in Asymptomatic Patients: A Meta-analysis and Systematic Review of the Literature
Source: Ann Surg Oncol. 2020 Nov 9;28(7):3751–60. doi: 10.1245/s10434-020-09313-9 (PMC8184724; doi:10.1245/s10434-020-09313-9)
Supplement: Supplementary file 1 — Supplementary material 1 (DOCX 12 kb) [file 10434_2020_9313_MOESM1_ESM.docx]

Supplementary table 1: Search terms for NAF cytology papers

| 1. Nipple Aspirate Fluid  2. ((Breast* or Nipple*) adj (secretion* or Aspirat*)).mp. [mp=title, abstract, original title, name of substance word, subject heading word, floating sub-heading word, keyword heading word, organism supplementary concept word, protocol supplementary concept word, rare disease supplementary concept word, unique identifier, synonyms]  3. 1 or 2  4. Cytodiagnosis  5. cytolog*.mp. [mp=title, abstract, original title, name of substance word, subject heading word, floating sub-heading word, keyword heading word, organism supplementary concept word, protocol supplementary concept word, rare disease supplementary concept word, unique identifier, synonyms]  6. papanicolaou.mp. [mp=title, abstract, original title, name of substance word, subject heading word, floating sub-heading word, keyword heading word, organism supplementary concept word, protocol supplementary concept word, rare disease supplementary concept word, unique identifier, synonyms]  7. cytodiagnos*.mp. [mp=title, abstract, original title, name of substance word, subject heading word, floating sub-heading word, keyword heading word, organism supplementary concept word, protocol supplementary concept word, rare disease supplementary concept word, unique identifier, synonyms]  8. 4 or 5 or 6 or 7  9. 3 and 8 |
| --- |
