# Supplementary material for: Diagnostic Accuracy of Nipple Aspirate Fluid Cytology in Asymptomatic Patients: A Meta-analysis and Systematic Review of the Literature
Source: Ann Surg Oncol. 2020 Nov 9;28(7):3751–60. doi: 10.1245/s10434-020-09313-9 (PMC8184724; doi:10.1245/s10434-020-09313-9)
Supplement: Supplementary file 2 — Supplementary material 2 (PDF 33 kb) [file 10434_2020_9313_MOESM2_ESM.pdf]

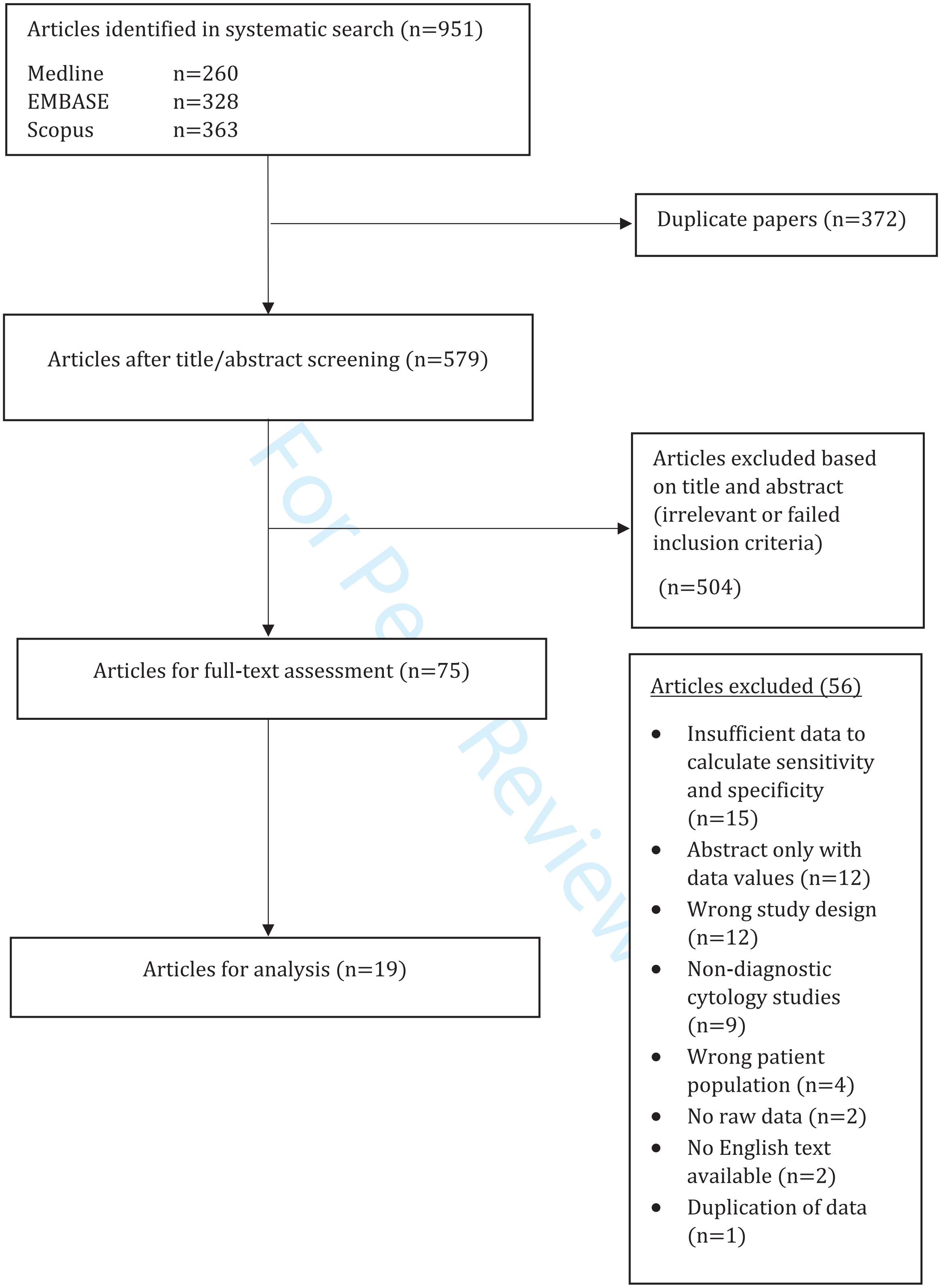

For Peer Review

Supplementary Figure 1 PRISMA flow diagram. Flow chart of studies included in the analysis (diagnostic cytology)
